# Exercise: The ultimate treatment to all ailments?

**DOI:** 10.1002/clc.23369

**Published:** 2020-06-07

**Authors:** Alexandros Kasiakogias, Sanjay Sharma

**Affiliations:** ^1^ Cardiology Clinical and Academic Group St George's University of London London UK

**Keywords:** cardiovascular disease, fitness, physical activity, sedentary

## Abstract

Extensive clinical research has provided robust evidence that exercise is a cost‐effective measure to substantially alleviate the burden of a large number of diseases, many of which belong to the cardiovascular (CV) spectrum. In terms of cardiac benefit, the positive effects of exercise are attributed to improvements in standard risk factors for atherosclerosis, as well as to its positive impact on several pathophysiological mechanisms for CV diseases. For secondary prevention, exercise, optimally in the context of a cardiac rehabilitation program, has been shown to improve functional capacity and survival. Clinicians should encourage physical activity and provide exercise recommendations for all patients, taking into consideration any underlying pathology. In the present review, the benefits of exercise for the prevention and treatment of major CV risk factors and heart conditions are analyzed.

## 1 INTRODUCTION

The notion that exercise is important for well‐being, health, and longevity has been documented throughout history and all over the world.^1^ Susruta, an Indian physician who lived during 600 Before Common Era (BCE) was the first healer who prescribed exercise and advised that the regimen should be performed daily and be of half the extent of the patient's capacity. Hippocrates (460‐370 BCE) identified the joined importance of food and exercise and was the first physician to prescribe exercise in written form for a patient “suffering from consumption.” Bernardino Ramazzini, the father of occupational medicine, noted that professional messengers were generally healthier compared to those with sedentary jobs such as tailors. Since the seminal studies from the 1950s, a burgeoning amount of evidence has accumulated, supporting exercise as a highly cost‐effective measure for the prevention and treatment of disease. Currently, exercise has been shown to be capable of preventing over 35 chronic conditions, many of which are within the cardiovascular (CV) spectrum, as well as of reducing CV and all‐cause mortality.^2^

Despite the world‐wide strategies to promote sufficient physical activity (PA) at a population level, adoption rates of a physically active lifestyle have been far from ideal. It is estimated that one in five adults lack acceptable PA and that only half the population in developed countries follow the widely applied recommendation of a minimum of 150 minutes of moderate‐intensity exercise weekly.^3^

Physical inactivity is a principal contributor to the global burden of disease. Calculations of population‐attributable fractions have shown that PA not meeting current recommendations is the cause of 6% of the burden of coronary artery disease (CAD), 7% of type 2 diabetes mellitus (T2DM), and 9% of premature death.^4^ Exercise may protect against certain cancers such as breast, colon, prostate, and pancreatic cancer. It also reduces rates of osteoporosis and hip fractures, dementia, anxiety and depression. The importance of exercise for CV protection is supported by mostly epidemiological data in the setting of primary prevention and randomized clinical study data in the setting of secondary prevention.^5^ In the present article, the current evidence of the associations of exercise with CV outcome will be reviewed. The benefits of exercise for the prevention and treatment of major CV risk factors and conditions will be analyzed.

## 2 TERMINOLOGY AND CLASSIFICATION BASICS

A number of different terms related to PA have been examined in clinical research relating to CV and non‐CV outcomes (Table 1).^2^ Sedentarism refers to prolonged periods of minimal energy expenditure associated with common practices such as television viewing and time on the computer. PA is any leisure or occupational activity that is accompanied by an increase in energy expenditure compared to the resting state. Exercise is PA that is planned and structured with the goal of promoting fitness and health. PA and exercise lead to improvements in physical and cardiorespiratory fitness (CRF), an effect that is also modulated by genetic factors.^7^

**TABLE 1:** Terminology related to physical activity^2^, ^6^

Term	Definition	Examples/components
Sedentary behavior	Waking behavior with energy expenditure ≤1.5 metabolic equivalents of task while in seated, reclined, or lying posture.	Television viewing, computer use, video games, lying in bed, sleeping, etc.
Physical activity	Any bodily movement produced by the contraction of skeletal muscles, which increases energy expenditure above the basal level of the resting state.	Household and occupational activities; commuting; gardening; dancing; exercise and sports, etc.
Physical inactivity	Physical activity levels less than those recommended for optimization of health and prevention of premature disease and death.	‐
Exercise	A subset of physical activity that is repetitive, planned, structured, and often goal‐oriented, intending to promote physical fitness and health.	Aerobic and endurance exercise; and resistance exercise.
Exercise training	A subsidiary of exercise that denotes physical activity performed during leisure time, targeting the improvement or maintaining of physical fitness and performance.	‐
Physical fitness	An individual's attribute resulting from physical activity and exercise and denoting the ability to carry out standard physical tasks without undue fatigue, and sufficient energy to enjoy leisure‐time activities and respond to emergencies.	Muscular strength; power, and endurance; cardiorespiratory endurance and fitness; flexibility; balance; speed of movement; and reaction time.
Sports	An activity involving physical exertion, coordination, and skill that is mastered through planned and vigorous training and is performed for improvement in physical fitness, entertainment or competition.	Power, endurance, skill, and mixed.
Athlete	An individual performing regular exercise training (for an arbitrarily defined volume of at least 4 to 5 h a week) in one or more sports disciplines in order to participate in competitive events at an amateur or professional level.	Power, endurance, skill, and mixed.

Classification of exercise is largely based on the principal form of energy production, the extent of body movement, the participating muscles, and the main benefitting component of physical fitness.^6^ Dynamic or isotonic exercise refers to the purposeful activity with repetitive movement of large muscles groups and joints that leads to substantial elevations in heart rate and expenditure of energy (eg, jogging, skiing, bicycling, and swimming). Static or isometric exercise is the contraction of muscles without movements of the joints. Resistance (strength) exercise is performed against an opposing force in order to improve muscular strength and/or endurance. It may be dynamic with changes in the length and tension of muscles (eg, weight‐lifting, circuit training, and elastic resistance bands) or static (eg, handgrip and plank position). Exercise sessions and their load are defined by their duration, frequency, intensity, and weekly volume. Intensity of exercise is traditionally defined by percentage of peak heart rate or peak oxygen consumption. For ease of patient communication, mild activity would allow singing, moderate activity would allow speaking, and vigorous activity would allow only a few words to be exhaled (the so‐called talk test). An exercise session is defined as aerobic or anaerobic based on the availability of oxygen for production of energy required for muscle contraction. Energy expenditure is generally expressed as Metabolic Equivalent of Task (MET) where 1 MET is energy expenditure at rest and equates to a peak oxygen consumption of 3.5 mL/min/kg. Energy expenditure of 3‐5.9 MET defines moderate‐intensity exercise and of >6 METs defines vigorous exercise. A proposed method to quantify aerobic exercise volume is to calculate the MET score by multiplying the frequency and duration per week of exercise by the intensity of exercise in MET. Current guidelines recommend approximately 7.5 MET‐hours/week of exercise.^8^

The most studied training modality is continuous exercise training, characterized by predominantly dynamic, moderate‐intensity exercise at a constant workload for a prolonged period of time. This modality has been shown to effectively improve maximum work capacity and reduce mortality in health and CV disease. High‐intensity interval training (HIIT) has been derived from standard athletic training programs and has emerged as one of the fastest growing exercise programs in recent years. A typical session consists of four to six repeated, short (1‐4 minutes) bouts of vigorous effort interspersed with brief periods (1‐3 minutes) of active recovery of moderate effort. High‐IIT may be more effective compared to continuous training in improving CRF, reducing weight and improving glycaemic control but its long‐term prognostic effect in CV disease needs further study. Pyramid training, characterized by stepwise increase and decrease of workload, is another interesting modality that may improve physical fitness in the shortest time, but also warrants further evaluation. Finally, resistance training is gaining ground as it improves muscle strength and aerobic capacity, with the maximum effect seen when it is combined with endurance exercise. Resistance training is particularly important for improving muscle tone, bone density, and coordination which become increasingly important with senescence.

## 3 PA AND OUTCOME BENEFITS

Forty years of thorough research has examined the epidemiological effects of PA on CV prognosis and mortality. Unsurprisingly, sedentarism/physical inactivity on one side and exercise/CRF on the other exhibit opposite associations with CV outcomes, including heart failure (HF) and CAD, and mortality (Table 2).^9^, ^10^, ^11^, ^12^, ^13^

**TABLE 2:** Representative studies published in the last 5 years examining associations of physical activity and fitness with mortality

Study	N	Design	Follow‐up (years)	Participants	Assessment of activity	Main finding
Arem et al (2015)^9^	661 137	Pooled analysis of six prospective studies	14	Men and women aged 21‐98 y	Study‐specific PA questionnaires.	Compared to no leisure PA, mortality risks were as follows: *<7.5 MET‐h/week*: HR, 0.80 (95% CI, 0.78‐0.82) *7.5‐15 MET‐h/week*: HR, 0.69 (95% CI, 0.67‐0.70) *15‐22.5 MET‐h/week*: HR, 0.63 (95% CI, 0.62‐0.65).
Lear et al (2017)^10^	141 945	Multinational prospective cohort study	7	Individuals aged 35‐70 y free of CV disease	1‐week total physical activity assessed using the long‐form International Physical Activity Questionnaire.	Compared to low PA (<150 min), combined mortality and major CV disease risks were as follows: *150‐750 min*: HR, 0.85 (95% CI, 0.80‐0.91) *>750 min*: HR, 0.73 (95% CI, 0.68‐0.77).
O'Donovan et al (2017)^11^	63 591	Pooled analysis of 11 cohorts from household‐based surveillance studies	18	Males and females older than 40 y	Self‐reported PA.	Compared with inactive participants, all‐cause mortality risks were as follows: *Insufficiently active*: HR, 0.66 (95% CI, 0.62‐0.72) *Regularly active*: HR, 0.65 (95% CI, 0.58‐0.73) *Weekend warriors*: HR, 0.70 (95% CI, 0.60‐0.82).
Imboden MT et al (2018)^12^	4137	Observational study (BALL ST cohort)	24	Self‐referred apparently healthy adults (42.8 ± 12.2 y).	Measurement of peak VO_2_ by cardiopulmonary exercise testing using standardized treadmill exercise protocols.	Each single increase in METs was associated with 11.6%, 16.1%, and 14.0% reductions in all‐cause, CV, and cancer mortality, respectively.
Clausen et al (2018)^13^	5107	Observational study (Copenhagen Male study)	46	Middle‐aged, employed men free of CV disease (40‐59 y)	Calculation of peak VO_2_ with Åstrand's nomogram by using a standard bicycle ergometer test.	After multivariate adjustments, compared to below normal CRF, added years of life expectancy were as follows: *Low normal CRF*: 2.1 y (95% CI, 0.7‐4.4; *P* = .002) *High normal CRF*: 2.9 y (95% CI, 1.5‐4.2; *P* < .001) *Above normal CRF*: 4.9 y (95% CI, 3.1 to 6.7; *P* < .001).

Abbreviations: CRF, cardiorespiratory fitness; CV, cardiovascular disease; MET, metabolic equivalents; PA, physical activity.

### 3.1 Sedentarism

A remarkably consistent association of sedentary behavior with all‐cause and CV mortality and CV disease has been documented. A recent meta‐analysis of 34 studies and over 1.3 million participants revealed a nonlinear relationship between total sedentary behavior and all‐cause and CV mortality independent of PA.^14^ Television viewing time presented the strongest association. A threshold of 6 to 8 hours/day of total sitting time and 3 to 4 hours per day of TV viewing over which the risk increased was identified. The calculated risk for each additional hour of TV viewing below and above 4 hours was 1.02 (0.99‐1.04) and 1.08 (1.05‐1.12), respectively. A linear dose‐response association of both average sedentary time and sedentary bout duration was identified with the use of accelerometers in 5638 older women (mean age of 79 years) free of CV disease participating in the Objective Physical Activity and Cardiovascular Health study.^15^ Women who were classified as having a high sedentary time and long durations of sedentary bouts had a 34% greater risk for CV disease (HR = 1.34; CI = 1.08‐1.65) compared to the reference group.

It is clear that an individual may be physically active, perhaps even following the current exercise recommendations, and still be sedentary for prolonged periods either at work or during leisure time. It has been estimated that half of the time spent awake is characterized by sedentary behavior. Recent data, however, suggest that concurrent increasing levels of PA may positively alter the association of sedentarism with a worse outcome. In a meta‐analysis that stratified patients by quartiles of PA, there was a dose‐response association between sitting time and CV mortality in the lowest PA quartile (9%‐32% higher risk), but this association was progressively ameliorated and eventually disappeared across increasing levels of PA.^16^ Current guidelines propose reducing the time spent sitting by introducing periods of PA of any kind.^17^ For people incapable of indulging in regular PA, alternating sitting periods with periods of standing is an efficient method for improving outcomes.^18^

### 3.2 PA and exercise

PA and exercise are independently associated with lower CV morbidity and CV and all‐cause mortality in a dose‐response manner. All types of PA are efficient in preventing CV disease irrespective of gender, age, and race and CV risk factors. Clinical guidelines of major societies recommend a minimum of 150 min/week of moderate intensity (1000 kcal/week) or 75 min/week of vigorous intensity aerobic exercise, with an optimal benefit in terms of longevity and protection from CV events expected in three to five times as much for either.^5^, ^8^, ^9^

Little exercise is better than none and even simple standing exhibits a dose‐response relationship with lower rates of mortality.^18^ Similarly, moderate or vigorous exercise that is below the recommended volume by current guidelines is still associated with lower rates of CV and all‐cause mortality. There is a curvilinear relationship between aerobic exercise volume and CV risk, where at the lower end of PA levels even small increases lead to large reductions in risk, while at the higher end there is a progressive decrease in the amount of benefit achieved.^9^

Exercise inevitably improves CRF, which is optimally evaluated by measurement of peak VO2 during cardiopulmonary exercise testing but may also be assessed by exercise time, maximum power output and other exams such as the 6‐minute walk test. CRF is actually a stronger predictor of outcome compared to PA or exercise training, as evident in studies in healthy populations and populations with or at risk of CV disease.^19^ A decrease in CV and all‐cause mortality risk ranging from 10% to 25% per 1 MET increase in exercise capacity has been observed in healthy individuals, and those with multiple risk factors or established CV disease and independently of gender, age or race.^12^, ^13^, ^20^ The association between CRF and mortality may be curvilinear, as is the case with exercise.

## 4 RISK FACTORS

**FIGURE 1:**
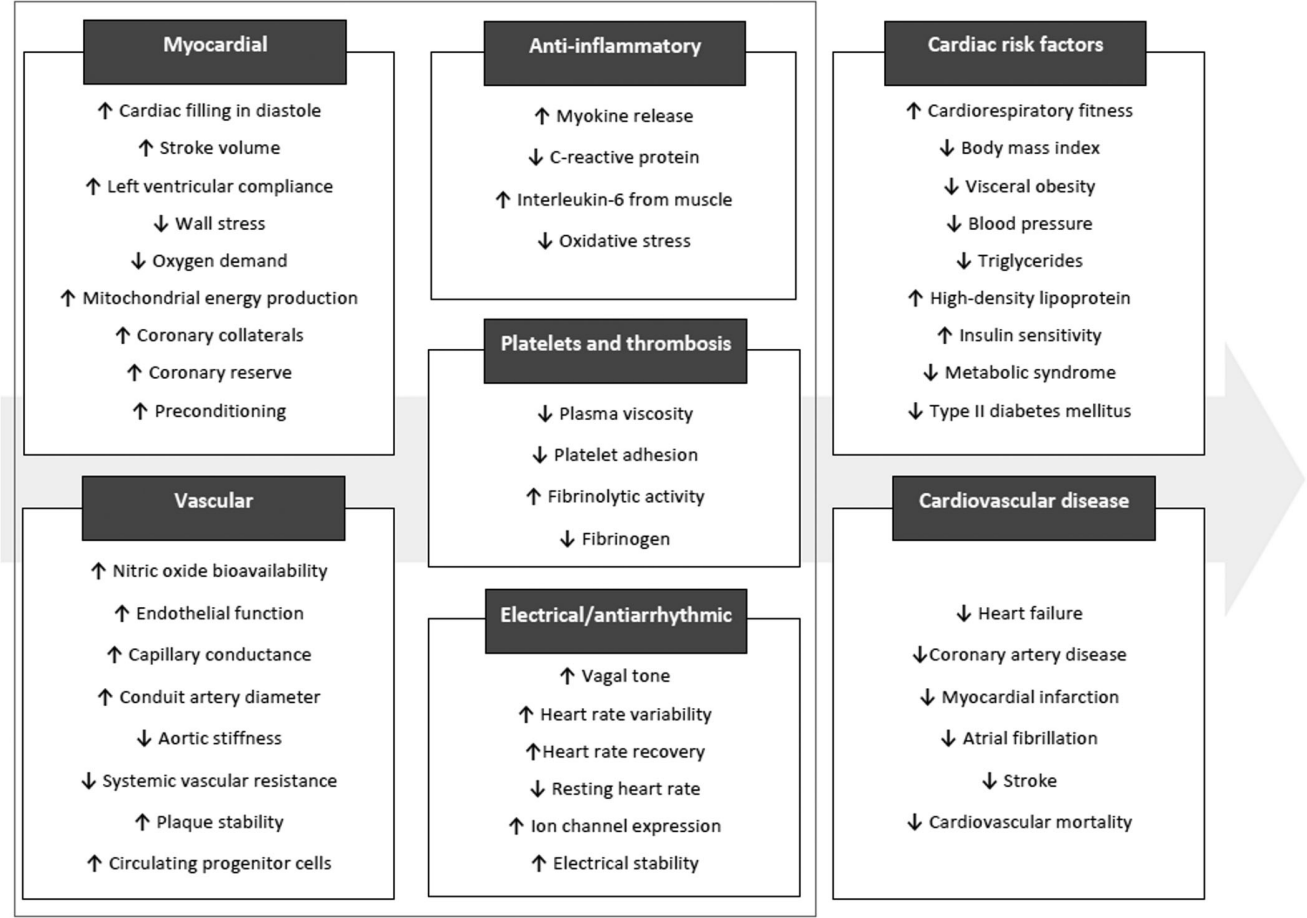
Cardiovascular effects of exercise. Regular exercise is accompanied by diverse beneficial effects in virtually all pathways associated with atherosclerosis and cardiovascular diseases. Reduction in primary and secondary cardiovascular risk is achieved primarily by improvements in classic risk factors, but also through alternate mechanisms such as autonomic modulation and anti‐inflammatory protection

The beneficial effect of exercise on CV outcome is primarily through modulation of standard CV risk factors for atherosclerosis including hypertension (HTN), diabetes, dyslipidemia, and obesity.^21^ More than two‐thirds of the expected risk reduction related to exercise seems to arise from improvements in these classic risk factors (Figure 1). In addition, individuals who exercise regularly would be expected to be leading a healthy lifestyle that includes abstaining from smoking. An acute bout of exercise has been shown to effectively reduce the urge of smoking in both men and women.^22^ The benefits of exercise on traditional CV risk factors are described below.

### 4.1 Hypertension

The effectiveness of exercise in reducing BP is evident from the phenomenon of acute postexercise hypotension that may last for up to 24 hours. In turn, chronic reduction in BP in response to exercise training has been well‐studied and attributed predominantly to improvements in nitric oxide‐mediated endothelial function and attenuations in systemic vascular resistance, and reduced activity of the sympathetic nervous system and the renin‐angiotensin‐aldosterone axis.^23^ An impressive number of large‐scale epidemiological studies, controlled clinical trials and subsequent meta‐analyses have consistently shown that regular aerobic exercise is accompanied by a BP‐lowering effect of 3 to 7 mmHg and potentially reaching 15 mmHg.^23^ The response of BP to exercise is greater with higher exercise volumes and perhaps more intense exercise and is more pronounced in those with a higher baseline BP. Indeed, the benefit of aerobic training is more evident in hypertensive individuals, and rivals the antihypertensive effects of commonly prescribed first‐line drugs. Accordingly, current relevant recommendations advise for an ideal of 300 minutes of moderate or 150 minutes of vigorous activity per week for patients with HTN or high normal BP.^24^ Twenty‐four‐hour BP evaluations in hypertensive individuals reveal that aerobic training has a greater impact on daytime rather than night time systolic BP.^25^ In patients with resistant HTN, an aerobic exercise program has been shown to decrease ambulatory BP by 6/3 mmHg.^26^

Isometric and resistance exercise were previously approached with skepticism in hypertensive patients due to fear of an exaggerated BP response, increases in arterial stiffness and further deterioration of HTN. In recent years, it has been shown that these are safe exercise modalities for hypertensive patients that also lower BP but perhaps to a lower extent compared to aerobic training. However, a meta‐analysis of 64 controlled studies of moderate‐intensity dynamic resistance training in patients with pre‐HTN showed a BP‐lowering effect comparable to aerobic exercise, and also identified a dose‐response effect that was greater in individuals with a higher resting BP and in populations who were not taking antihypertensive drugs.^27^ In another large meta‐analysis of 93 randomized controlled trials attempting a head‐to‐head comparison of the effects of different types of exercise, it was shown that endurance, dynamic resistance, and isometric resistance training lower both systolic and diastolic BP, with the effect being more evident for isometric exercise; however, the conclusions were based on a relatively small number of studies including isometric exercise.^28^ Resistance exercise is recommended two to three times per week in the current HTN guidelines, but these recommendations may be refined in the future with more accumulating evidence.^24^

Regular exercise is also effective in preventing HTN, with some data supporting that higher exercise loads are associated with lower rates of developing HTN. In a meta‐analysis of 29 studies on over 3000 normotensives, it was shown that the risk of HTN decreased by 6% for every 10 METs of task hours/week increase in leisure‐time PA.^29^ In the same study a similar risk reduction was observed in individuals meeting the standard exercise recommendations compared to inactive participants. Along these lines, a hypertensive BP response to exercise has been associated with a higher risk of HTN, target organ damage, and a worse CV outcome. Regular aerobic exercise and increased fitness have been shown to ameliorate this phenomenon in various populations including treated hypertensive patients.^30^

It is self‐explanatory that the estimated BP‐lowering effect of exercise in patients with HTN is also associated with significant improvements in CV outcomes. A decrease in BP of 6 to 7 mmHg would translate to a CV risk reduction of up to 20% to 30%. Accordingly, exercise capacity has been associated with lower mortality in both prehypertensive and hypertensive individuals. Even among older hypertensive individuals, every 1‐MET increase in exercise capacity is associated with an 11% lower risk of death.^31^

### 4.2 Lipid metabolism

Aerobic exercise has been associated with a beneficial effect on lipoprotein levels in both normolipidemic and hyperlipidemic individuals, even though the overall effect is small and concurrent weight loss may have contributed in the positive findings of some of the relevant studies. Beneficial effects have been noted mostly for high‐density lipoprotein‐cholesterol (HDL‐C) levels but also for low‐density lipoprotein‐cholesterol (LDL‐C) particle size and apolipoprotein B and triglyceride levels.^32^ The combination of exercise with dietary changes provides greater reductions in LDL‐C compared to either measure alone. Exercise volume rather than intensity seem to be better associated with lipid changes. It has been estimated that the minimal exercise volume in order to increase HDL‐C level is 900 kcal of energy expenditure or 120 minutes of exercise per week.^33^ The Studies of Targeted Risk Reduction Interventions through Defined Exercise (STRRIDE) was a randomized controlled study that examined the effects of 7 months of two different volumes and intensities of aerobic exercise on risk factors in overweight and obese individuals with mild and moderate dyslipidaemia.^34^ All exercise programs showed evidence of beneficial changes in the lipoprotein profile. The high‐volume high‐intensity group showed the greatest decreases in small LDL‐C without affecting the total LDL‐C concentration. The same amount of exercise led to increases in HDL‐C cholesterol as well as in decreases in triglycerides. In terms of outcome, among dyslipidaemic individuals with a mean age of 58 years, statin treatment and increased physical fitness have been independently associated with lower mortality, while combined statin treatment and high fitness levels were associated with a better prognosis compared to either intervention alone.^35^

### 4.3 Insulin sensitivity and diabetes mellitus

Regular leisure‐time activity of different intensities has been associated with a 25% to 40% reduction in the risk of T2DM.^17^ Similarly, each 1 MET higher CRF has been associated with an 8% lower risk of T2DM.^36^ Habitual PA has been consistently associated with improved insulin sensitivity and responsiveness, improved lipid profiles and BP levels as well as decreases in glycated hemoglobin (HbA1c) by 0.5% to 0.7% in prediabetic and diabetic patients. A meta‐analysis of 56 studies on such patients showed that an increase of 100 minutes in PA per week was associated with an average decrease in fasting glucose by 4.71 mg/dL and in HbA1c by 0.16%.^37^ An analysis of the Health Benefits of Aerobic and Resistance Training in Individuals With Type 2 Diabetes trial showed that even patients with no change in their CRF after 9 months of exercise revealed significant improvements in metabolic parameters including decreases in HbA1c.^38^ Similarly, in patients with the metabolic syndrome, aerobic exercise is accompanied by improvements in all of its components, including body mass and waist circumference, BP, and lipid profile.^39^ Taking just 5‐minute breaks of prolonged sitting by standing or walking is an effective way to elicit metabolic benefits in populations at high risk of diabetes. For this reason, The American Diabetes Association recommends that sedentary behavior should be interrupted by periods of light intensity PA.^17^

A single session of low intensity exercise leads to acute improvements in insulin sensitivity and uptake of fatty acids from skeletal muscles. However, the benefits are more substantial with longer periods and more intense bouts of exercise. An earlier meta‐analysis has shown that a 20‐week program of moderate‐intensity aerobic exercise improves CRF and glycated hemoglobin (HbA1c), the latter decreasing in parallel with more intense exercise.^40^ Resistance training has also been shown to decrease HbA1c, increase lean body mass and improve the response to the glucose tolerance test. A meta‐analysis of 14 trials in a total of 915 patients with T2DM that examined the effects of aerobic, resistance and combined training documented an overall better response of HbA1c, fasting glucose, and triacylglycerols to combined training compared to the other modalities alone.^41^ Current guidelines thus recommend that adults with T2DM should follow an exercise training program that includes both aerobic and resistance exercises to improve insulin metabolism and reduce all‐cause and CV mortality.^17^

### 4.4 Obesity

Regular PA protects against weight gain throughout life, maintains long‐term weight loss and preserves lean body mass during caloric restriction.^42^ Long‐term exercise also seems to result in larger reductions in predominantly abdominal fat.^43^ Nevertheless, meta‐analyses that have examined the effects of exercise on weight loss have shown that both high or low intensity exercise were associated with only modest reductions in weight (by about 1.5 Kgr).^44^ Even when added to caloric restriction the additional effect of exercise on weight loss remains limited. Of note, it has been shown that CRF modulates the association of weight with mortality, and obese but fit individuals exhibit similar survival to individuals with normal weight.^45^

## 5 CV CONDITIONS AND SECONDARY PREVENTION

In the setting of secondary prevention, multiple studies have shown that PA and exercise as well as CRF reduce the risk of new CV events. Exercise may be performed at home, in community facilities or, ideally, in the context of a cardiac rehabilitation program.^5^ The latter provides the foundations for structured exercise prescription combined with lifestyle advice and psychosocial support. Resistance exercise should be included in the training routine in order to maintain muscle mass and strength, further reduce body fat, increase CRF and improve quality of life.^46^

### 5.1 Coronary artery disease

Lack of PA is an independent predictor for CAD development. In a case control study evaluating multiple CV risk factors for acute myocardial infarction (MI) across 52 countries, regular exercise was associated with a 14% lower risk of MI among men and women of all age groups and ethnicities.^47^ Conversely, lack of PA accounted for 12% of the population‐attributable risk of an acute event. More recently, in a meta‐analysis of 33 studies, a dose‐response relationship of PA and CAD was noted; a 20% risk reduction in individuals engaging in 300 minutes per week of moderate‐intensity activity was documented.^48^

A number of benefits of exercise in patients with established CAD have been observed. Regular exercise contributes to greater functional capacity and higher thresholds of angina presentation. At the myocardial level, the effects of exercise may include retardation of disease progression and regression of coronary plaques, improvements in endothelial function, favorable mechanisms for healing endothelial damage, coronary collateralization, and a positive effect on left ventricular function and remodeling.^49^ Following MI, exercise training exerts the greatest benefits on ejection fraction and left ventricular volumes when started as early as 1 week after the acute episode and lasting longer than 3 months.^50^

With respect to hard endpoints, there is consistent evidence that exercise in the context of cardiac rehabilitation programs is associated with lower CV mortality in patients after MI or after coronary revascularisation. In a large meta‐analysis of 63 studies examining variable intensities and periods of aerobic exercise as part of cardiac rehabilitation in patients post‐MI or revascularization, a 26% reduction in CV death and an 18% reduction in hospital admissions was documented.^51^ In contrast, data on such benefits in patients with stable CAD are limited by the small number of studies and small number of participants and a review of 7 studies and 581 patients with stable CAD failed to show an additional benefit of exercise‐based cardiac rehabilitation regarding mortality, acute events, or hospitalisations.^52^ Interestingly, some data have shown that in patients with CAD, high levels of PA may be harmful. An analysis of patients with stable CAD after an acute event or revascularisation procedure documented a reverse J‐shaped association of PA with CV mortality over a 10‐year period.^53^ Patients who performed 5 times the exercise recommendations showed more than a double risk of CV mortality compared to those active two to four times per week. Therefore, individuals with CAD who aspire to perform high‐volume or intensity of exercise should be evaluated thoroughly to exclude inducible myocardial ischaemia, left ventricular dysfunction, and exercise related arrhythmias.^54^

### 5.2 Heart failure

Historically, exercise was prohibited in patients with HF due to fear of haemodynamic compromise. Since the 1980's, a large set of evidence has been accumulated that supports exercise as a strong preventive, prognostic, and therapeutic modality in HF both in the setting of primary and secondary prevention. PA is associated with a lower risk of HF across a wide range of body weights and independently of comorbidities.^55^ A large meta‐analysis of 12 prospective studies confirmed an inverse, linear, dose‐response relationship between PA and risk of HF. PA as per current guidelines (150 minutes of moderate‐intensity exercise per week) was associated with a 10% lower risk of HF compared to no activity, while exercise levels double and quadriple of the guidelines were associated with a 19% and 35% lower risk, respectively.^56^ Similarly, a number of studies in different populations has shown that for each added MET of greater CRF there is an approximately 20% lower risk of future HF.^57^ A report of the Coronary Artery Risk Development in Young Adults study showed that lower CRF at a young age is associated with abnormal remodeling of the left ventricle and subclinical systolic and diastolic abnormalities in middle age.^58^ Recent evidence suggest that the protective effect of exercise and CRF may be greater for HF with preserved ejection fraction (HFpEF) than HF with reduced EF (HFrEF). A report from data derived from three cohort studies documented a dose‐response association between leisure‐time PA and HFpEF but not HFrEF.^59^

In patients with established HF, the lack of PA has been shown to be accompanied by a doubling of risk of all‐cause and CV mortality.^60^ Exercise intolerance and low CRF affect both quality of life and overall prognosis in these patients, particularly in the elderly, where frailty and cardiac and noncardiac comorbidities are much higher. In cases with HFrEF, exercise also improves resting ventricular volumes, peak exercise cardiac output and cardiac reserve, peak oxygen consumption, and the lactate threshold.^61^ Indeed, exercise in patients with either HFrEF or HFpEF improves peak VO2 by approximately 3 mL/Kg or, in other words, by 15% to 17%, that would translate in a mortality reduction of approximately 30%.^62^ In the very heterogenous population with HFpEF, things are less clear, as, for instance, it is well‐established that long‐term exercise may protect from the age‐related stiffening but not the impairment of ventricular relaxation.^63^ However, the role of diastolic dysfunction in the response of HFpEF patients to exercise training remains to be elucidated.

Most early studies of exercise in HF were small and single‐centered and were based on continuous moderate‐intensity aerobic training. Altogether, they have provided promising evidence that exercise training is accompanied by improvements in quality of life and decreases in the risk of hospitalisations and long‐term mortality. An earlier meta‐analysis of the ExTraMATCH Collaborative has shown that exercise training for at least 8 weeks reduces mortality by 45% in patients with HFrEF.^64^ The more recent HF: A Controlled Trial Investigating Outcomes of Exercise Training trial is the largest and most cited randomized controlled trial in the field and examined the clinical benefit of a supervised followed by an at‐home aerobic training regimen on top of standard optimal therapy in patients with HFrEF of New York Heart Association class II to IV and mean age of 59 years.^65^ This landmark study was plagued by low (30%) patient adherence but did document a mildly significant decrease in the risk of CV mortality and HF hospitalization by 15%, observable after prespecified adjustment for major prognosticators. Accordingly, an updated meta‐analysis by the Cochrane Collaboration Group reported that cardiac rehabilitation in HF patients decreases hospitalisations in the short‐term 12 months) and may improve all‐cause mortality in the long term.^66^ Cardiorespiratory exercise training is recommended by all major HF guidelines and it is promising that the number of countries offering dedicated exercise training programs for these patients is increasing.^67^

### 5.3 Atrial fibrillation

A number of studies have shown that regular PA and satisfactory CRF are associated with a lower incidence of atrial fibrillation (AF), a finding at least partially mediated by concurrent improvements in established risk factors for AF such as HTN and obesity. In numbers, moderate PA in individuals aged more than 65 years reduces the risk of AF by 44%.^68^ The protective effect of exercise for AF may disappear and actually reverse with long‐term, high‐volume endurance training. Atrial dilatation, pulmonary vein stretch and induction of profibrotic pathways have been suggested as potential mechanisms for this observation. Of note, atrial enlargement with normal or supranormal atrial mechanics is considered to be a feature of the athlete's heart that results from increased cardiac output and volume overload and is not associated with a higher prevalence of supraventricular arrhythmias.^69^

In patients with AF, exercise is safe, as long as sufficient rate control has been achieved, and it has been shown to improve AF‐related symptoms, exercise tolerance, and quality of life. Exercise may also have a favorable effect on AF burden and AF recurrence, while more study data are needed regarding the optimal training regimen as well as the effects on hard endpoints in these patients.^70^ More recently, in a randomized controlled trial in patients with either paroxysmal or persistent AF, 12 weeks of aerobic HIIT was associated with significant improvements in time in AF, exercise capacity and left atrial and ventricular function.^71^

## 6 CAN TOO MUCH EXERCISE HARM THE HEART?

There are emerging studies reporting a J‐shaped relationship between exercise dose and CV morbidity and mortality. Some studies have also shown there are diminishing returns beyond an exercise capacity of 12 METs.^72^, ^73^ Small studies in lifelong endurance athletes have revealed evidence of coronary artery calcification (CAC) in almost 1 in 5 master male athletes and myocardial fibrosis in approximately 1 in 10 master male athletes.^74^ Coupled with an increased prevalence of AF, there is a possibility that the stresses of lifelong intensive exercise may have a potentially deleterious effect in some individuals with a previously normal heart in male athletes, as the same has not been reported in master female athletes. Short‐ and medium‐term studies have failed to show any association with increased CAC and CV mortality but larger prospective studies are required to understand why one man's high may represent another man's poison.^75^

## 7 CONCLUSIONS

The strong assets of regular moderate exercise are its free availability at low cost and its simultaneous benefits for preventing and improving several cardiac diseases. Integration of exercise in the daily routine of all individuals is essential to improve global health. It is important to emphasize that current evidence suggest that exercising for three to five times the current recommendations is the ideal dose for maximum benefit to prevent several CV diseases, although even 1 hour per week of exercise is associated with a health benefit. Some individuals engaging in lifelong endurance exercise show a high prevalence of CAC, myocardial fibrosis and AF, the long‐term significance of which is uncertain. Overall regular exercise is associated with a significant reduction in CV morbidity and mortality and longevity of an additional 2 to 7 years of life.

## CONFLICT OF INTEREST

The authors declare no potential conflict of interests.
